# Molecular Pathogenesis of Secondary Acute Promyelocytic Leukemia

**DOI:** 10.4084/MJHID.2011.045

**Published:** 2011-10-24

**Authors:** M. Joannides, A. N. Mays, A. R. Mistry, S. K. Hasan, A. Reiter, J. L. Wiemels, C. A. Felix, F. Lo Coco, N. Osheroff, E. Solomon, D. Grimwade

**Affiliations:** 1 Department of Medical & Molecular Genetics, King’s College London School of Medicine, UK; 2 Department of Biopathology & Fondazione Santa Lucia, University of Tor Vergata, Rome, Italy; 3 III.Medizinische Klinik, Universitätsmedizin Mannheim, Mannheim, Germany; 4 Department of Epidemiology and Biostatistics, University of California, San Francisco, San Francisco, USA; 5 Division of Oncology, Children’s Hospital of Philadelphia, Philadelphia, USA; 6 The Departments of Biochemistry and Medicine, Vanderbilt University School of Medicine, Nashville, USA

## Abstract

Balanced chromosomal translocations that generate chimeric oncoproteins are considered to be initiating lesions in the pathogenesis of acute myeloid leukemia. The most frequent is the t(15;17)(q22;q21), which fuses the *PML* and *RARA* genes, giving rise to acute promyelocytic leukemia (APL). An increasing proportion of APL cases are therapy-related (t-APL), which develop following exposure to radiotherapy and/or chemotherapeutic agents that target DNA topoisomerase II (topoII), particularly mitoxantrone and epirubicin. To gain insights into molecular mechanisms underlying the formation of the t(15;17) we mapped the translocation breakpoints in a series of t-APLs, which revealed significant clustering according to the nature of the drug exposure. Remarkably, in approximately half of t-APL cases arising following mitoxantrone treatment for breast cancer or multiple sclerosis, the chromosome 15 breakpoint fell within an 8-bp “hotspot” region in *PML* intron 6, which was confirmed to be a preferential site of topoII-mediated DNA cleavage induced by mitoxantrone. Chromosome 15 breakpoints falling outside the “hotspot”, and the corresponding *RARA* breakpoints were also shown to be functional topoII cleavage sites. The observation that particular regions of the *PML* and *RARA* loci are susceptible to topoII-mediated DNA damage induced by epirubicin and mitoxantrone may underlie the propensity of these agents to cause APL.

## Introduction

Acute myeloid leukemia (AML) is characterized by a spectrum of recurring chromosomal abnormalities, which distinguish biologically and prognostically distinct subtypes of disease (reviewed 1). To date, more than one hundred balanced chromosomal rearrangements (translocations, insertions and inversions) have been identified and cloned,^2^ with evidence suggesting that these are critical initiating events in the pathogenesis of AML. Identification of the genes involved in chromosomal rearrangements has provided major insights into the regulation of normal hematopoiesis and how disruption of key transcription factors and epigenetic modulators promote leukemic transformation. A number of genes, including *MLL* at 11q23, *NUP98* at 11p15, *RARA* at 17q21 and *RUNX1* at 21q22, are recurrent targets of chromosomal rearrangements in AML, being fused to a range of potential partner genes (reviewed 3). Interestingly, chromosomal rearrangements involving these particular loci also have been noted as a feature of secondary acute leukemias arising as a complication of prior therapy involving drugs targeting topoisomerase II (topoII), which are widely used in the treatment of a variety of tumors.^4^–^13^ TopoII is a critical enzyme that relaxes supercoiled DNA and removes knots and tangles from the genome by transiently cleaving and religating both strands of the double helix via the formation of a covalent cleavage intermediate (reviewed 14). Epipodophyllotoxins (e.g. etoposide), anthracyclines (e.g. epirubicin) and anthracenediones (e.g. mitoxantrone) act as topoII poisons, inducing DNA damage by disrupting the cleavage-religation equilibrium and increasing the concentration of DNA topoII covalent complexes, which leads to apoptosis of the tumor cells.^14^

The association between exposure to chemotherapeutic agents targeting topoII and development of leukemias with balanced chromosomal rearrangements has naturally implicated the enzyme in this process, but the mechanisms involved have remained subject to debate. One hypothesis takes into account reports that leukemia-associated translocations can be detected in hematopoietic cells derived from healthy individuals without overt leukemia,^15^,^16^ suggesting that administration of chemotherapy and/or radiotherapy provides a selective advantage to progenitors with pre-existing translocations during regrowth of depopulated bone marrow. Moreover, the exposure to DNA damaging agents could lead to the acquisition of additional mutations that cooperate with the chimeric fusion protein generated by the translocation to induce leukemic transformation. A second hypothesis, based on findings with transformed cells, raised the possibility that agents targeting topoII could lead to the formation of chromosomal translocations through an indirect mechanism involving induction of apoptotic nucleases.^17^–^20^ Interestingly, Rolf Marschalek and colleagues provide evidence for a third potential mechanism, showing that the region of the *MLL* locus where breakpoints associated with infant and therapy-related leukemias cluster, colocalize with an internal promoter element, highlighting the relevance of chromatin structure and DNA topology in the genesis of chromosomal translocations.^21^ Finally, the fourth hypothesis, which is based on increasing biochemical and genetic evidence, suggests that in the presence of a topo II-targeting chemotherapeutic agent, topoII plays a direct role in generating double-stranded DNA breaks in regions of the genome that are particularly susceptible due to the nature of the surrounding chromatin. Following aberrant repair, these breaks go on to generate leukemia-associated chromosomal translocations (reviewed 22).

Intriguingly, the nature of the drug exposure has a bearing on the molecular phenotype of the resultant secondary leukemia, with translocations involving the *MLL* gene at 11q23 being particularly associated with etoposide exposure.^10^,^23^,^24^ Development of therapy-related acute promyelocytic leukemia (t-APL), characterized by the t(15;17)(q22;q21), has been linked to treatment with mitoxantrone and epirubicin.^12^,^25^,^26^ The t(15;17) leads to fusion of the gene encoding the myeloid transcription factor RARα (Retinoic Acid Receptor Alpha) at 17q21 with a gene that was previously unknown - designated *PML* (for ProMyelocytic Leukemia), which has subsequently been found to be involved in growth suppression and regulation of apoptosis (reviewed 27). This subtype of leukemia is of particular interest, being the first in which molecularly targeted therapies (i.e., all-*trans*retinoic acid [ATRA] and arsenic trioxide [ATO]) have been successfully used in clinical practice.^27^ These agents act by inducing degradation of the PML-RARα oncoprotein, leading to clinical remission and have resulted in dramatic improvements in clinical outcome (reviewed 28). They also offer a potentially curative approach in patients with t-APL who have already received significant doses of chemotherapy for their previous condition and may be close to the anthracycline ceiling, or who are considered unfit for conventional therapy.^29^

The majority of t-APL cases arise in patients who have undergone treatment for breast cancer, where mitoxantrone and epirubicin have been widely used.^12^,^25^,^26^,^30^ In this setting, cumulative doses of epirubicin of ≤720mg/m^2^ have been associated with a secondary leukemia risk of 0.37% at 8 years.^31^ As more patients survive their primary cancers, secondary leukemias are becoming an increasing healthcare problem.^32^ Although t-APL remains relatively uncommon, two case series have suggested that the incidence has risen in recent years, with up to 20% of APL patients presenting with secondary disease.^25^,^30^

## Investigation of Molecular Mechanisms in Mitoxantrone-Related t-APL

**Figure 1:**
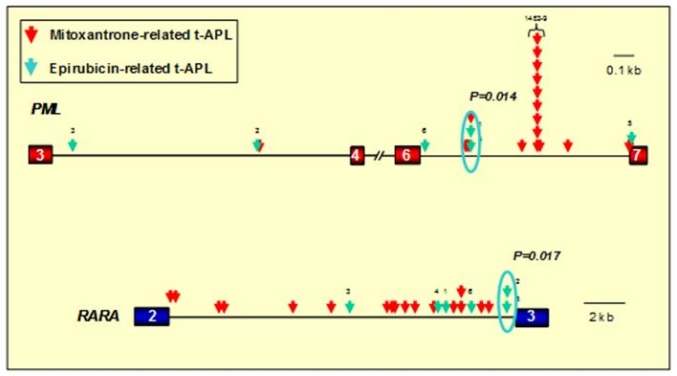
Distribution of translocation breakpoints within the *PML* and *RARA* loci in t-APL cases arising following epirubicin and mitoxantrone. *PML* exons are represented by red boxes, *RARA* exons in blue and introns are represented by black lines. Arrows indicate the location of *PML* and *RARA* translocation breakpoints identified by long-range PCR and sequence analysis in patients with t-APL arising following mitoxantrone (red arrows) or epirubicin (green arrows). In 12 patients mitoxantrone was used for treatment of multiple sclerosis (MS). In the remaining 5 patients with mitoxantrone-related APL and the 6 patients with t-APL following epirubicin, these agents were used for prior breast cancer. Significant breakpoint clustering was observed, with a “hotspot” identified in *PML* intron 6 (position 1482–9) in mitoxantrone-related APL (following use of the drug for MS or breast cancer) and separate clusters associated with APL arising following epirubicin exposure. Chromosomal breakpoints were confirmed to be preferential sites of drug-induced topoisomerase II cleavage in functional assays (see Figure 2). Adapted from Mays et al.^42^ with permission.

**Figure 2:**
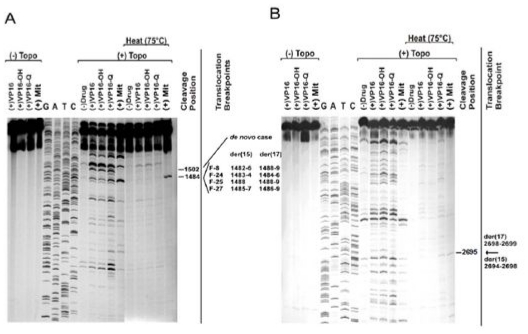
Demonstration of mitoxantrone-induced topoisomerase II dependent DNA cleavage at translocation breakpoints in therapy-related APL. **A)** *In vitro* DNA topoisomerase II (topoII) cleavage assay carried out for a *PML* substrate containing the breakpoints of 4 treatment-related APL (t-APL) cases (F-8,-24,-25,-27) within the 8-bp breakpoint “hotspot” (positions 1482–1489). Patients received combination chemotherapy including the topoII poison mitoxantrone for breast cancer. Control reactions were carried out in the absence of topoII (lanes 1–4), and in the presence of etoposide (VP16), etoposide catechol (VP16-OH), etoposide quinone (VP16-Q) and mitoxantrone (Mit). Dideoxy sequencing reactions of the substrate are shown in lanes 5–8. Cleavage reactions were carried out by exposure to human topoIIα in the absence of drug (lane 9), and in the presence of etoposide (lane 10), etoposide catechol (lane 11), etoposide quinone (lane 12) and mitoxantrone (lane 13). Additional cleavage reactions were carried out to evaluate the heat-stability of cleavage complexes formed by incubation at 75°C for 10 min (lanes 14–18). The nucleotide shown by the dash is the 5′ side of the cleavage site (-1 position), which corresponds to the der(15) and der(17) translocation breakpoints in 4 cases of mitoxantrone-related APL (far right). The cleavage site at position 1484 was observed in the absence of drug, and in the presence of etoposide, both etoposide metabolites and mitoxantrone (lanes 9–13). Cleavage at this position was the strongest site observed in the presence of mitoxantrone (lane 13). Furthermore, the complexes formed at this site were shown to be heat-stable in the presence of mitoxantrone (lane 18). Interestingly, a cleavage site at position 1502 is also observed, which corresponds to a breakpoint detected in a case of *de novo* APL. **B)** TopoII cleavage assay of normal homologue of der(15) and der(17) *RARA* translocation breakpoints in APL of one of the mitoxantrone-related cases (F-8). The substrate spanning positions 2603 to 2871 of *RARA* intron 2 contained the translocation breakpoints. Dash at right shows (−1) position of cleavage site corresponding to der(15) and der(17) translocation breakpoints (arrow far right). Adapted from Mistry et al.^33^ with permission.

As a first step to gain insights into mechanisms underlying formation of the t(15;17) we used long-range polymerase chain reaction (PCR) and sequence analysis to define chromosomal breakpoint locations, comparing the pattern between patients presenting *de novo* (n=35) and those with t-APL occurring following exposure to mitoxantrone (n=6) or other agents/radiation therapy (n=7).^33^ Analysis of diagnostic samples from large cohorts of patients with *de novo* APL has established that the majority of chromosome 15 breakpoints fall within 3 breakpoint cluster regions (bcr) i.e. in intron 3 (bcr3), intron 6 (bcr1) and exon 6 (bcr2) of the *PML* locus, accounting for approximately 40%, 55% and 5% of cases respectively.^34^ Chromosome 17 breakpoints fall within the ~17kb intron 2 of the *RARA* locus, such that the PML-RARα fusion retains key functional domains mediating DNA binding and interaction with coactivator/corepressors, retinoid-X-receptor and ligand (i.e. retinoic acid).^27^ While breakpoints observed in *de novo* APL appeared broadly distributed, chromosome 15 breakpoints of each of the mitoxantrone-related t-APLs fell within *PML* intron 6, with 4 cases clustering within an 8-bp region (position 1482–9)(see Figure 1).^33^ Given that this intron is over 1kb in length, this clustering of breakpoints was highly unlikely to have occurred by chance (p<0.001 by scan statistics). To investigate this further, we used a functional *in vitro* assay, in which substrates containing the normal homologues of the *PML* and *RARA* translocation breakpoints are 5′-end-labelled and exposed to clinically relevant topoII poisons (e.g. mitoxantrone, epirubicin, etoposide) in the absence or presence of human topoII alpha; cleavage complexes are trapped and cleavage sites mapped in relation to the observed translocation breakpoints at the sequence level.^35^–^37^ These experiments demonstrated that the breakpoint “hotspot,” identified in t-APL patients previously treated with mitoxantrone for breast cancer, corresponded precisely to a preferential mitoxantrone-induced topoII-dependent DNA cleavage site at position 1484 (see Figure 2).^33^ Moreover, each observed translocation breakpoint within the *RARA* locus on chromosome 17 was confirmed to be a preferred site of topoII-mediated DNA damage induced by mitoxantrone (Figure 2).^33^

These data strongly implicated mitoxantrone in the etiology of t-APL. However, it is important to consider that the study of patients developing leukemia following cancer therapy presents a challenge, given that they have often been exposed to multiple cytotoxic drugs in addition to radiotherapy. This makes it difficult to identify the causative agent with any certainty. Moreover, such patient populations could feasibly be enriched for individuals at particular risk of leukemia, having already developed one form of cancer. Therefore, to provide further insights into molecular mechanisms in topoII-related leukemias, we analyzed a cohort of 12 patients collected from across Europe who developed APL following the use of single agent mitoxantrone to treat a benign condition, multiple sclerosis (MS), and in whom there was no history of previous cancer.^38^ Chromosome 15 breakpoints again were found to cluster at position 1484 within *PML* intron 6. Furthermore, shared chromosome 17 breakpoints that were preferential sites of mitoxantrone-induced topoII cleavage in functional assays were identified within *RARA* intron 2 (Figure 1).^38^ The series of mitoxantrone-related t-APL cases analyzed has been further extended recently, with the chromosome 15 breakpoint found to fall within the “hotspot” region in 12 of 23 cases (52%).^33^,^38^,^39^

**Figure 3:**
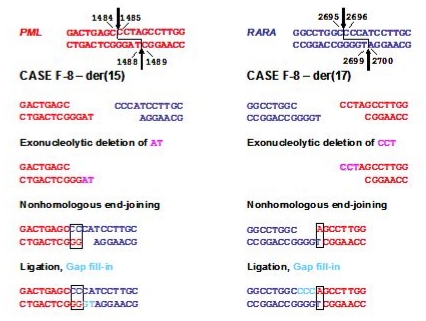
Model for formation of the t(15;17) in a case of mitoxantrone-related t-APL (case F8) following topoII induced cleavage in *PML* and *RARA* loci with 4-base 5′ overhangs and aberrant DNA repair. Native *PML* and *RARA* sequences are red and blue, respectively. The processing includes exonucleolytic nibbling to form two-base (der(15)) or single-base (der(17)) homologies and creation of both breakpoint junctions by error-prone nonhomologous end-joining (NHEJ). In formation of the der(15), positions 1487–1488 on the antisense strand of *PML* are lost by exonucleolytic nibbling (pink) before NHEJ joins the indicated bases. Positions 1485–1487 on the sense strand of *PML* are lost by exonucleolytic nibbling (pink) and the der(17) forms by NHEJ. Template-directed polymerization of the relevant single-stranded overhangs fills in any gaps (light blue). Each *RARA* overhang is preserved completely. Adapted from Mistry et al.^33^ with permission.

**Figure 4:**
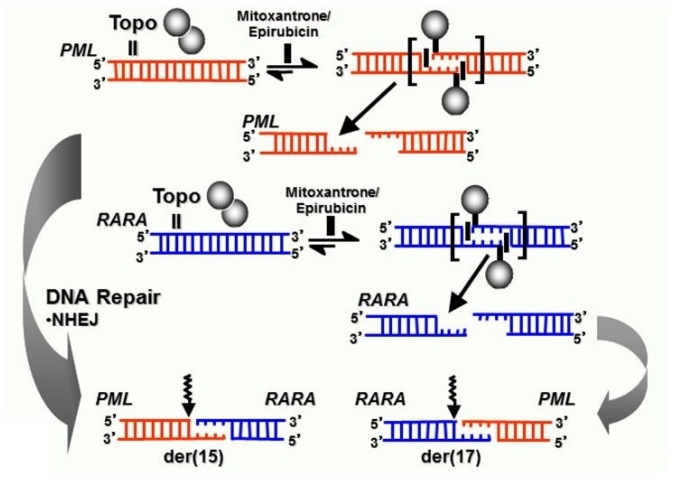
Model summarizing formation of reciprocal translocation breakpoint junctions in treatment related APL directly by generation of drug-stimulated topoisomerase II cleavage complexes and near-precise or precise NHEJ repair. Adapted from Felix et al.^22^ with permission.

Comparison of the genomic breakpoint junction regions with the native genes showed that translocations in mitoxantrone-related t-APL were reciprocal, generally without loss or gain of nucleotides.^33^,^38^ Presence of sequence homologies between *PML* and *RARA* suggests that topoII-mediated DNA damage may be repaired by the canonical nonhomologous end-joining (NHEJ) or the alternative end-joining (alt-NHEJ) pathway, which require minimal overlapping sequences between nonhomologous chromosomes to repair double-stranded DNA breaks (reviewed^40^). Using the information derived from genomic breakpoint junction sequence analysis and *in vitro* cleavage assays, the knowledge that topoII introduces staggered nicks in DNA with 4-bp overhangs^22^ and considering known mechanisms of DNA repair it was possible to construct models by which the t(15;17) may have been formed in each case analyzed (see Figures 3 & 4). Taken together, these data provide very strong evidence that mitoxantrone is a causative agent in the pathogenesis of t-APL.

## Investigation of Molecular Mechanisms in t-APL Cases Arising Following Other TopoII Poisons

Epirubicin exposure has been linked to secondary leukemias with a range of balanced rearrangements, including translocations involving the *MLL* locus, core binding factor leukemias and t-APL with the t(15;17).^31^,^41^ In order to gain further insights into molecular mechanisms underlying epirubicin-related leukemias, we characterized t(15;17) genomic breakpoint junction regions in a series of 6 t-APL cases that arose following breast cancer therapy.^42^ Epirubicin was generally used as a component of combination chemotherapy, with a median latency period from first exposure to presentation of t-APL of 26 months (range 18–48 months). Although the number of cases examined was small, significant breakpoint clustering was observed in both the *PML* and *RARA* loci (P= .009 and P = .017, respectively), with *PML* breakpoints lying outside the mitoxantrone-associated “hotspot” region (Figure 1). Functional assays demonstrated that recurrent breakpoints identified in the *PML* and *RARA* loci in epirubicin-related t-APL were preferential sites of topoII-induced DNA damage that were enhanced by epirubicin.^42^ Again, using the same approach as for mitoxantrone-related t-APLs, models could be constructed to explain the formation of the t(15;17) in APLs arising following epirubicin exposure.^42^

There also have been reports of t-APL occurring following treatment with other topoII poisons (e.g., etoposide) used for lymphomas and various solid tumors, as well as Langerhans cell histiocytosis.^12^,^25^,^43^ To determine whether topoII–mediated cleavage is relevant to other drugs associated with t-APL, we also have studied a patient in whom APL developed after treatment for laryngeal carcinoma that included etoposide and doxorubicin.^33^ Etoposide and its metabolites and doxorubicin induced topoII to cleave DNA at the *PML* and *RARA* translocation breakpoints. Moreover, the cleavage sites could recombine to form the der(15) and der(17) breakpoint junctions observed in this patient. Taken together, these results suggest that topoII–mediated cleavage is a general mechanism causing DNA damage in APL that develops after treatment with various agents that target topoII (Figure 4).

## Concluding Remarks

While therapy-related leukemias are still relatively uncommon, they are characterized by the same range of cytogenetic abnormalities that are found in cases of AML arising *de novo*.^44^,^45^ Indeed, greater understanding of therapy-related leukemias may provide significant insights into the biology of their *de novo* counterparts. For example, defining the latency period between first exposure to a leukemogenic agent (e.g. mitoxantrone) and presentation with full blown leukemia, provides clues to the timeframe between acquisition of chromosomal rearrangements such as the t(15;17) and progression to leukemia in the *de novo* setting. Analysis of t-APL cases suggests that the median time to develop APL from the formation of the t(15;17) is ~27 months,^33^,^38^,^42^ implying the need for cooperating mutations. While logistically challenging, therapy-related leukemias afford the opportunity for tracking the stepwise acquisition of mutations that are required for progression to full-blown leukemia,^46^,^47^ and which may be of relevance to the pathogenesis of leukemias arising *de novo*. While we have observed a few cases of t-APL that present within 12 months from first mitoxantrone exposure, latency periods in the majority of cases are much longer. This may account for why, even if the t(15;17) were acquired in some cases *in utero*, *de novo* pediatric APL only very rarely presents in infancy.

A number of studies conducted over the last three decades have served to identify specific dosing schedules or particular agents that are associated with high rates of induction of secondary leukemias,^32^,^48^–^51^ leading to the development of effective alternative treatment protocols that are substantially safer.^52^–^54^ However, the study of patients with t-APL has demonstrated that therapy-related leukemias also can occur in patients subject to very low level exposure, as exemplified by a case of APL involving the *PML* breakpoint “hotspot” arising following a single 15 mg dose of mitoxantrone used as adjuvant chemotherapy for breast cancer.^33^ Epidemiology studies conducted in MS patients treated with mitoxantrone have suggested that the risk of development of secondary leukemia is ~1 in 370, 55,56 with the majority of reported cases being t-APL. This raises key questions as to the extent to which the play of chance is involved in the development of therapy-related leukemias, as well as the relative importance of patient predisposition to the development of this complication. A number of prerequisites have to be satisfied to develop leukemia following treatment with a topoII poison. Firstly, double-strand DNA breaks must occur within two genes with the potential to form oncogenic fusions. These breaks then need to be repaired to generate in-frame functional chimeric fusion genes. This translocation event needs to occur in a progenitor permissive for leukemic transformation and finally the necessary cooperating mutations are acquired. While our studies have provided evidence that topoII plays a direct role in mediating DNA damage that leads to formation of the t(15;17) in t-APL, a key question remains as to whether the enzyme is also involved in the formation of chromosomal translocations in *de novo* leukemias. Exposure to environmental toxins and agents targeting topoII has been implicated in the development of infant leukemia with translocations involving *MLL* at 11q23.^57^–^59^ Interestingly, recent evidence lends further support for topoII in the etiology of chromosomal translocations, inducing DNA damage in the *TMPRSS2* and *ERG* loci in response to androgen signalling, leading to formation of fusion genes involved in prostate cancer.^60^

It is readily conceivable that genetic susceptibility to primary tumors due to mutations or functional variants for example in DNA repair pathways also could increase the risk of development of secondary leukemias. Interestingly, a recent genome-wide association study has implicated variants in the *PRDM1* gene at 6q21 in the development of second neoplasms in children treated with radiotherapy for Hodgkin’s disease,^61^ whereas whole genome sequencing applied in a case of therapy-related AML arising from early-onset BRCA1/2 mutation-negative breast and ovarian cancer revealed a novel TP53 cancer susceptibility mutation.^62^ The spectrum of resultant leukemias could reflect the nature of the genetic susceptibility as well as the agents preferentially used for the particular primary condition, as would be suggested by the propensity of etoposide to induce secondary leukemias involving the *MLL* gene at 11q23 and epirubicin and mitoxantrone to induce t-APL. Moreover, genetic variation in the handling of a range of specific cytotoxic agents could affect an individual’s risk of developing secondary leukemia (reviewed 63). Indeed, it has recently been reported that genetic variants of genes encoding drug-metabolizing enzymes and components of DNA repair pathways are associated with increased susceptibility to development of t-APL in patients with MS receiving mitoxantrone.^64^

Dissecting out the relative importance of these factors represents a considerable challenge. It requires the analysis of substantial patient cohorts, which are well characterized both in terms of their primary tumors, prior cytotoxic therapy and cytogenetic and molecular profile of the secondary leukemias. Nevertheless, significant progress in this research area is likely to be fruitful allowing not only the development of more individualized and safer approaches to treatment of primary tumors, but also (potentially) providing insights into molecular mechanisms underlying the pathogenesis of *de novo* leukemias. Thus, it could afford improved understanding of AML as a whole.
